# Pregnancy and early motherhood among adolescents in five East African countries: a multi-level analysis of risk and protective factors

**DOI:** 10.1186/s12884-019-2204-z

**Published:** 2019-02-06

**Authors:** Yohannes Dibaba Wado, Elizabeth A. Sully, Joyce N. Mumah

**Affiliations:** 1 African Population and Health Research Centre, APHRC Campus, Manga Close, P.O. Box 10787-00100, Nairobi, Kenya; 2 Guttmacher Institute, 125 Maiden Lane, New York, NY 10038 USA

**Keywords:** Adolescents, Pregnancy and motherhood, East Africa, Multi-level analysis

## Abstract

**Background:**

Adolescent pregnancy remains a major challenge in both developed and developing countries. Early and unintended pregnancies among adolescents are associated with several adverse health, educational, social and economic outcomes. The aim of this study was to identify the contextual factors that influence adolescent pregnancy and early motherhood in five East African countries.

**Methods:**

We use DHS data from five East African countries to examine trends and risk factors associated with adolescent pregnancy. DHS surveys collect detailed information on individual and household characteristics, sexual behavior, contraception, and related reproductive behaviors. Our analysis focuses on a weighted subsample of adolescent’s age 15–19 years (Kenya, 5820; Tanzania, 2904; Uganda, 4263; Malawi, 5263; Zambia, 3675). Multilevel logistic regression analysis was used to identify the net effects of individual, household and community level contextual variables on adolescent pregnancy after adjusting for potential confounders.

**Results:**

Adolescent pregnancy and early motherhood is common in the five countries, ranging from 18% among adolescents in Kenya (2014) to 29% in Malawi (2016) and Zambia (2014). Although all five countries experienced a decline in adolescent pregnancy since 1990, the declines have been largely inconsistent. More than half of the adolescent’s most recent pregnancies and or births in these countries were unintended. The regression analysis found that educational attainment, age at first sex, household wealth, family structure and exposure to media were significantly associated with adolescent pregnancy in at least one of the five countries after adjusting for socio-demographic factors.

**Conclusion:**

The study highlights the importance of considering multi-sectoral approaches to addressing adolescent pregnancy. Broader development programs that have positive impacts on girls educational and employment opportunities may potentially influence their agency and decision-making around if and when to have children. Likewise, policies and programs that promote access to and uptake of adolescent sexual and reproductive health services are required to reduce barriers to the use of adolescent Sexual and Reproductive Health (SRH) services.

## Background

Although improving adolescent sexual and reproductive health is high on the global health agenda, adolescent pregnancy remains a major challenge in both developed and developing countries. Each year an estimated 21 million pregnancies occur among adolescent girls of age 15–19 years in developing countries, almost half of which (49%) are unintended [1, 2]. This results in an estimated 16 million births and more than 3.2 million abortions annually. In sub-Saharan Africa (SSA), an estimated 45% of the pregnancies among young women age 15–19 are unintended resulting in unintended births, unsafe abortions and miscarriages [2]. Nearly half of the unsafe abortion cases in sub-Saharan Africa also occur among adolescent girls and young women under the age of 25 years [3]. In Kenya, for instance adolescent girls make up nearly 17% of post abortion cases and account for 45% of severe abortion related complications treated in health facilities [4].

Early and unintended pregnancies among adolescents are associated with several adverse health, educational, social and economic outcomes. Childbirth is risky for adolescent girls and research indicates that pregnancy-related conditions are the second major cause of death among adolescent girls in developing countries [5, 6]. Adolescent pregnancy also interrupts young women’s schooling, thus endangering their future economic opportunities, including reducing job market opportunities [7, 8]. The effects of adolescent childbearing also extends to the health of their infants with evidence of higher perinatal deaths and low birth weight among babies born to mothers under 20 years of age [5, 6, 9, 10].

Adolescents in sub-Saharan Africa have one of the highest birth rates compared to adolescents in the other regions of the world, accounting for a significant proportion of the overall fertility in many countries in the region [11]. High adolescent birth rates reflect the vulnerabilities they experience and the lack of opportunities available to them [12, 13]. A wide range of factors contribute to the high rates of unintended pregnancy and unplanned births among adolescents; Poor knowledge of sexual and reproductive health services, legal barriers to accessing services, provider bias, stigma around premarital sex and lower decision making autonomy of married adolescents are among the factors contributing to unintended pregnancies [14–16].

Several studies examined the risk and protective factors associated with adolescent pregnancy using an ecological perspective, thus identifying multidimensional factors at the individual, relational, familial and structural levels that influence adolescent pregnancy. Among the several factors at the different levels, systematic reviews showed that poverty, and lower educational attainment are consistently associated with adolescent pregnancy [17–19]. Poverty, lack of education and gender inequality perpetuate early age at marriage, early childbearing and undermine successful transition from adolescence to adulthood. Poverty and socio-economic deprivation expose adolescent girls to early sexual debut and intergenerational sex [20, 21] and research indicates that adolescents from families of low socio-economic status and living in poor neighborhoods are at greater risk of early and unintended pregnancies largely due to poverty and lower expectations of future economic success [21, 22]. While the effects of socio-economic disadvantage and neighborhood poverty on early adolescent pregnancy is relatively well documented in the setting of developed countries, few studies examined their influences on adolescent child bearing in the setting of sub-Saharan African countries. Evidence of the influences of educational attainment on delaying adolescent pregnancy and early motherhood is also relatively well documented [17, 23, 24].

Apart from socio-economic disadvantage, systematic reviews show that disrupted family structure is also consistently related to adolescent pregnancy [17, 25]. Parent/child closeness or connectedness, parental supervision or regulation of children’s activities, and parents’ values against adolescent intercourse decrease the risk of adolescent pregnancy through adolescents remaining sexually abstinent, postponing intercourse, having few sexual partners or using contraception more consistently [25].

While body of research on the determinants of adolescent child bearing exist both in developed and developing countries, few studies have focused on the combination of individual, household and community-level factors in Eastern Africa. Adolescent pregnancy rates are high in the Eastern African region, necessitating a deeper examination into the determinants of adolescent pregnancy and childbearing. Understanding the correlates of adolescent pregnancy can motivate policy and programmatic responses to adolescent pregnancies and help monitor progress toward reducing their incidence. However, few studies examined the determinants of adolescent pregnancy and early motherhood in some of the countries where adolescent pregnancy is very high, such as those in Eastern Africa. The objective of this study is to identify the contextual factors that influence adolescent pregnancy in five east African countries.

## Methods

### Data

We use Demographic and Health Surveys (DHS) data from Kenya, Tanzania, Uganda, Malawi and Zambia to examine trends in adolescent pregnancy since 1989/1990 and identify the socio-economic and demographic correlates using a subsample of adolescents age 15–19 in the DHS data set. The DHS is implemented by the host country’s National Bureau of Statistics along with ICF International – a United States based organization that provides technical assistance to the DHSprogram. DHS is a nationally representative household survey with cross-sectional design and widely used for monitoring and evaluation of population, health and nutrition programs in developing countries.

The five countries included in the study all fall under the East Africa region geographically, have conducted five or more rounds of DHS for trend analysis and comparability and have relatively high level of adolescent pregnancy. The most recent surveys, all since 2013, were used to examine the key predictors of adolescent pregnancy and motherhood (Kenya 2014 with a weighted sample of 5820 adolescents; Uganda 2016 with a weighted sample of 4263; Tanzania 2015 with a weighted sample of 2904; Zambia 2014 with a weighted sample of 3675 and Malawi 2015 with a weighted sample of 5263 adolescents).

DHS surveys collect detailed information on individual and household characteristics, fertility, sexual behavior, contraception, and related reproductive behaviors from women of reproductive age (15–49 years). DHS follows a standardized procedure across countries for sampling, data collection, and data cleaning to allow for inter-country analysis. In all the surveys, a two-stage stratified sampling is employed. At stage one, regions/provinces/states are stratified into urban and rural areas. Enumeration areas or clusters from each region are sampled proportional to size. In sampled households, all women aged between 15 and 49 years who consent to participate in the survey are interviewed. Sample size for the surveys varied, both overtime for individual countries and between the countries included in the study.

### Variables

#### Outcome variable

The outcome variables of the study are self-reported current pregnancy or motherhood among adolescents, which refers to the proportion of adolescents age 15–19 who were either pregnant or had a live birth at the time of the interview. Our data are only current pregnancies or previous births and do not capture adolescent pregnancies that ended in miscarriage, abortion or stillbirth.

#### Explanatory variables

Although several previous studies used the ecological framework of multidimensional factors at the individual, relational, familial and structural levels that influence adolescent pregnancy, we could not use this framework fully due to the limitations in our data. The data we used (secondary data) is not collected to capture the constructs in the model comprehensively – and as a result we have focused on the influence of few constructs at the individual, household and community levels that we have available data for.

***Individual level covariates*** hypothesized to influence adolescent pregnancy include girl’s education, exposure to media, age, and age at first sex. The variable exposure to media is constructed from questions in the DHS on whether the respondent listens to radio, watches television, or reads newspapers and magazines. A categorical variable is created from these three questions measuring exposure to media; coded as 0 if she has no exposure to any of the three sources, 1 if she has exposure to one of the three sources, 2 if she has exposure to two of the three sources and 3 if she has exposure to all three sources. Age at first sex was recoded in to four categories: coded as 0 if she has not initiated sex, 1 if started sex before age 15, 2 if started sex between 15 and 17 years, and 4 if started sex between 18 and 19 years.

***Household level covariates*** include household wealth index, place of residence, sex of the household head and living arrangement (family structure). Household wealth index is coded into five categories in the DHS from the poorest to the richest. Place of residence is coded as 1 if urban and 2 for rural. Similarly, the variable on sex of the household head is coded as 1 for male and 2 for female. The variable for living arrangement comes from the DHS question on relationship to the head of the household and recoded into the following three categories; those living with their parents (a single parent), those living as married or cohabiting, and those living with relatives and non-relatives.

***Two contextual community level variables***, both created from the mean values at the cluster level, were included in the multivariate mixed effects model. These include cluster level household poverty and cluster level adolescent education. The cluster level poverty values were recoded as ‘low poverty’ for values above the mean and ‘high poverty’ for values below the mean. This is based on the hypothesized relationship between adolescent pregnancy and area deprivation**;** adolescent living in areas of poverty may be influenced by the low level of availability of services, poor living conditions, cultural practices of early marriage, sexual violence and related challenges. Likewise, poor people living in low poverty communities can also be thought to live in an empowered social and physical environment than those living in poverty-stricken communities. Cluster level girls education is created by aggregating the individual values of the educational attainment of adolescents at the cluster level and categorized in high and low levels based on the mean values.

### Ethical review and consent

The survey protocols of DHS data are reviewed by the ICF Institutional Review Board (IRB) and by Institutional Review Board in the host country. While the ICF IRB ensures that the survey complies with regulations for the protection of human subjects, the host country IRB ensures that the survey complies with laws and norms of the nation. The National Bureau of Statistics and Ministry of Health of the host countries coordinate the process of obtaining ethical approval from the national IRBs. In addition, an informed oral consent is obtained from each respondent and a parent or guardian provides consent for an adolescent under 18 years. The description of DHS consent process is available from https://www.dhsprogram.com/What-We-Do/Protecting-the-Privacy-of-DHS-Survey-Respondents.cfm.

### Data analysis

Data were analyzed using STATA statistical software version 14. First, we conducted exploratory analyses of each of the variables and conducted descriptive analysis looking at trends and patterns of adolescent pregnancy. Country-level bivariate analyses were conducted to examine associations between adolescent pregnancy and the selected individual, household and community level variables. Variables were included into the multivariate mixed effects logistic regression if they had a significant association at the bivariate level. Multi-level logistic regression analysis was used to identify the net effects of each explanatory variables after adjusting for potential confounders [26]. We report the fixed effects in terms of odds ratios (OR), and 95% confidence interval (CI) after adjusting for potential confounders.

One of the major drawbacks faced while examining cluster level variables was the small sample size within clusters after limiting the analysis to adolescents of age 15–19 years. The intra-cluster coefficient (ICC) was low for the empty model, suggesting less variation is due to the cluster level. Thus, we decided to keep only two of the contextual cluster level variables in the analysis, cluster level poverty and cluster level girls’ education.

## Results

Table 1 shows the socio-demographic characteristics of adolescents in the five target countries between the oldest and most-recent DHS surveys. As shown in the table, there has been great progress in terms of educational attainment, access to media, and knowledge of family planning among adolescents in the last two decades. Educational attainment has improved, with average years of schooling increasing in all five countries. Adolescents in Kenya (8.1 years) and Zambia (7.7 years) attained higher years of schooling on average than adolescents in the three other countries. Moreover, the proportion of adolescent girls with secondary and above education more than doubled in all the five countries between 1989 and 2016. In addition, higher proportion of adolescents resided in urban areas during the later DHSs (Table 1).

**Table 1:** Socio-demographic Characteristics of adolescents age 15–19 years during the earliest and latest years of DHS data in the five east African countries

Key characteristics	Kenya		Uganda		Tanzania		Malawi		Zambia	
	1989 (*N* = 1496)	2014 (*N* = 5820)	1989 (*N* = 1199)	2016 (*N* = 4263)	1991 (*N* = 2183)	2015 (*N* = 2904)	1992 (*N* = 1105)	2015 (*N* = 5263)	1992 (*N* = 1984)	2014 (*N* = 3675)
Mean years of schooling (mean, SD)	7.1 (2.4)	8.1 (2.6)	4.2 (2.9)	6.6 (2.7)	5.5 (2.7)	7.0 (2.7)	3.4 (3.0)	6.5 (2.6)	5.4 (2.6)	7.7 (2.5)
Secondary education (%)	21.4	47.8	12	33.5	6.4	35.1	4.5	27.2	24.2	59.6
Urban residence (%)	18.4	31.9	13.5	24.3	27.3	37.3	11.7	17.5	51.5	48.0
Richest households (%)	–	19.2	–	23.2	–	29.7	–	23.4	–	27.9
Exposure to media^a^ (%)	71.8	74.9	38.6	62.4	55.5	56.7	61.7	54.1	67.7	69.21
Ever married (%)	18.5	14.2	40.8	22.8	28.3	25.3	41.2	26.8	29.7	17.0
Sexually active (%)	46.1	37.2	62.3	45.7	51.4	52.2	–	51.9	61.0	49.1
Median age at first sex (median age)	15.0	15.2	14.7	15.5	15.3	15.4	–	15.5	15.0	15.0
Median age at 1st marriage (median age)	16.1	16.1	15.6	16.3	16.1	16.1	16.0	16.3	16	16.0
Knowledge of contraception (%)	80	96.4	73.2	96.7	51.4	94.2	75.5	93.6	76.8	95.3
Current use of contraception (%)	4.0	27.1	2	10.1	6.4	19.6	11.0	29.1	2.8	10.6

^a^Exposure to media was measured differently in the oldest surveys using questions on listening to radio every week / every day or watching TV every week/every day

Information on age at sexual debut and age at marriage was available for those who were sexually active and married before the time of the survey. Despite some significant increases in age at marriage and age at first sex over time, early marriage and early sexual debut are common. Over a quarter of adolescent girls of age 15–19 years in Tanzania and Malawi were married at the time of their most recent survey. At 14%, Kenya has the lowest proportion of adolescents ever married by age 19 years. Overall, all four countries have experienced a fall in the proportion of adolescents marrying before age 19, with the steepest decline observed in Uganda between 1989 and 2016 (Table 1). A higher proportion of the adolescent pregnancies and births occurred among adolescents in-union, ranging from 67% in Malawi and Uganda to 54% in Zambia during their most recent DHS (result not shown).

Early sexual debut, measured by the proportion of adolescents who have ever been sexually active, also declined in Kenya and Uganda, but increased slightly in Tanzania from 1991 to 2015. In Uganda, the median age at first sex and median age at first marriage has slightly increased between 1989 and 2016. (Table 1). Other important determinants of adolescent sexual and reproductive health, including knowledge of contraception, use of contraception and exposure to media, also improved in almost all the five countries over the same time period.

### Trends in adolescent pregnancy and motherhood

**Fig. 1:**
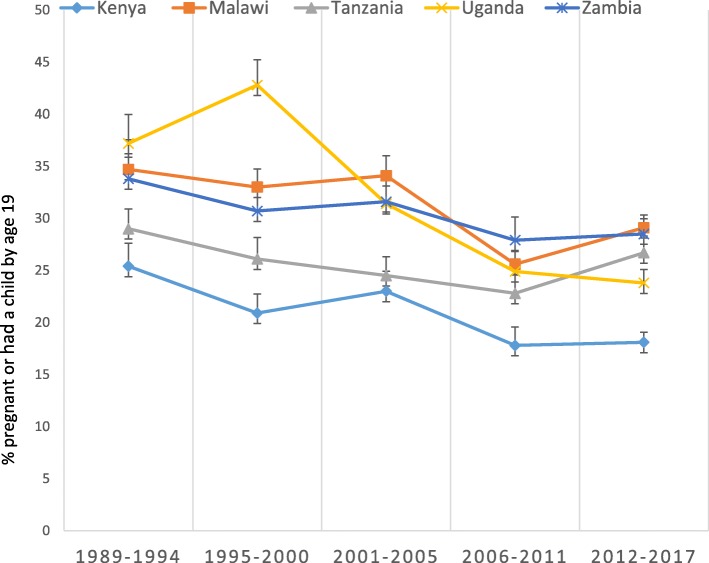
Trends in Adolescent Pregnancy and motherhood in 5 countries in East Africa, 1990–2017

Early motherhood is common in the five countries, ranging from 18% in Kenya (2014) to 29% in Malawi (2016) and Zambia (2014). In Tanzania and Uganda nearly 27 and 25% of adolescent girls age 15–19 were either pregnant or had their first child at the time of the most recent DHS respectively. Although all five countries have experienced a decline in adolescent pregnancy since 1990, the trends in the decline have been largely inconsistent. In Tanzania for instance, adolescent pregnancy declined from 29% [95% CI; 27.1–30.9] in 1989 to 23% in 2010 [95% CI; 21.1–24.6], but this trend recently was reversed increasing to about 26.7% [95% CI; 25.1–28.3] in 2015. In Uganda, adolescent pregnancy and motherhood increased initially but has consistently declined since then. Between 1989 and 2016, adolescent pregnancy and motherhood declined by 33% from 37.2% [95% CI; 34.4–40.0] to 24.8% [95% CI; 23.5–26.1], a significant decline compared to 1989 levels. Kenya, with the lowest level of adolescent pregnancy among the five countries, also reduced adolescent pregnancy from 25% [95% CI; 23.2–27.7] in 1989 to 18% [95% CI; 17.1–19.1] in 2014. Malawi also experienced a significant decline in adolescent pregnancy and motherhood since 1992, declining from 34.7% [95% CI; 31.9–37.5] in 1991 to 29% [95% CI; 27.8–30.3] in 2015. In Zambia, adolescent pregnancy declined very little since the first DHSs (Fig. 1).

Of the five countries, Tanzania and Malawi experienced an upward trend in teenage pregnancy during the latest surveys (In Tanzania it increased from 22.8% in 2010 to 26.7% with non-overlapping confidence intervals, and in Malawi from 25.6 to 29.1% during the same period). This has caused several policy level conversations and debates in the last few years.

Moreover, social inequalities in adolescent pregnancy and motherhood is evident in all five countries; by place of residence, age, educational attainment and wealth status among others. Across all the countries, the proportion of adolescents who had begun childbearing was higher in rural areas, among those with no education and amongst the poorest households. Variation is also observed with exposure to media, family structure and religion of respondents. In Zambia and Kenya, significant variation was observed in adolescent pregnancy and motherhood with the sex of the household head (Table 2).

**Table 2:** Percentage of adolescents who were either pregnant or begun child bearing by socio-demographic characteristics in five East African countries using the most recent DHS

Variables	Kenya	Malawi	Uganda	Tanzania	Zambia
	2014 (*N* = 5820)	2015 (*N* = 5263)	2016 (*N* = 4263)	2015 (*N* = 2904)	2014 (*N* = 3675)
Educational status					
No education	33.2^b^	54.1^b^	34.6^b^	52.3^b^	53.2^b^
Primary	23.7	32.2	28.7	33.8	35.8
Secondary & higher	11.5	18.4	16.7	10.4	23.0
Age, in years					
15	3.2^b^	4.5^b^	3.2^b^	4.4^b^	4.9^b^
16	8.0	12.2	9.4	11.4	11.9
17	15.0	26.6	22.1	23.0	25.7
18	25.9	45.6	40.2	38.2	41.7
19	39.9	59.2	53.9	56.7	58.9
Religion					
Catholic	18.3	25.5	22.9	–	23.7
Protestant	17.7	23.5	26.1		29.8
Muslim	17.5	38.2	26.5		4.0
Others	49.8	32.0	23.5		46.5
Exposure to media (newspaper, radio, TV)					
None	21.5^a^	31.5^a^	26.4^a^	32.9^a^	43.1^a^
One of the three	22.1	27.3	26.8	25.8	29.0
Two of the three	13.2	19.1	18.6	20.4	18.9
All three	6.5	11.1	13.1	10.7	10.7
Residence					
Urban	17.0^a^	21.3^b^	18.8^b^	18.5^b^	20.0^b^
Rural	18.4	30.7	26.7	31.6	36.4
Wealth index					
Poorest	26.0^b^	43.6^b^	33.5^b^	42.4^b^	44.5^b^
Poorer	18.3	34.8	31.9	38.5	38.6
Middle	19.1	30.5	24.6	28.2	34.5
Richer	16.4	24.7	21.5	23.4	28.2
Richest	10.2	15.3	24.8	12.7	10.3
Sex of the household head					
Male	19.5^a^	30.0	28.0^b^	27.6	29.0
Female	16.0	27.1	19.0	23.8	27.4
Family living arrangement					
Living with husband/partner	73.1^b^	84.6^b^	75.5^b^	80.5^b^	90.1^b^
Living with parents	8.7	11.6	12.0	14.7	20.1
Living with relatives or others	18.4	17.8	20.8	23.7	21.8
Total	18.1	29.0	24.8	26.7	28.5

^a^differences statistically significant at 5%, ^b^ differences statistically significant at 1%

Table 3 shows the proportion of adolescent current pregnancies and most recent births by intention status among adolescents in all the five countries. A high proportion of the adolescent’s most recent births and pregnancies were unintended, ranging from 61.7% in Kenya to 41.3% in Tanzania. In all the five countries, higher proportion of adolescents with secondary and higher education, younger adolescents, and adolescents living in urban areas reported that their most recent births and pregnancies were unintended compared to adolescents with no or lower education, older adolescents and those living in rural areas. Only in Kenya, the proportion of adolescent pregnancy and recent birth was higher among rural adolescents compared to their urban counterparts (Table 3).

**Table 3:** Percentage of adolescent’s recent pregnancies and births that were unintended in the East African countries, using the most recent DHS

Variables	Kenya, 2014 *n* = 481	Malawi, 2015 *n* = 1515	Uganda,2016 *n* = 1053	Tanzania, 2015 *n* = 771	Zambia, 2014 *n* = 1025
Educational status					
No education	8.8	29.9	41.6	21.4	34.0
Primary	60.8	49.9	52.4	40.0	46.8
Secondary & higher	68.6	65.3	62.0	65.4	66.8
Age, in years					
15	88.5	85.2	93.5	40.2	77.1
16	79.8	68.7	66.7	45.4	56.3
17	59.1	55.9	55.3	52.7	60.3
18	59.0	51.2	52.3	36.5	59.0
19	57.5	44.0	50.3	39.9	52.2
Residence					
Urban	56.0	51.9	56.1	57.0	69.2
Rural	64.5	51.6	53.9	35.9	50.5
Wealth index					
Poorest	52.6	43.6	52.4	28.4	40.3
poorer	69.5	34.8	53.2	36.9	50.9
Middle	64.0	30.5	58.6	46.0	65.9
Richer	68.2	24.7	47.9	49.4	62.3
Richest	48.8	15.3	61.9	57.5	76.5
Total (%)	61.7	51.6	54.3	41.3	56.8

### Multivariable multi-level analysis

Table 4 presents results of the multivariable multilevel logistic regression analysis by country. We included the following variables into the regression model: age, place of residence, religion, education, wealth, marital status, exposure to media, sex of household head, family structure, age at first sex, cluster level poverty and education. The regression analysis identified several contextual individual, household and community-level factors that are associated with adolescent pregnancy and motherhood in these countries after adjusting for socio-demographic factors of age, place of residence, religion, and marital status. Educational attainment, household wealth, family structure or living arrangement, exposure to media were significantly associated with adolescent pregnancy in at least one of the five countries. Education and household wealth status were consistently associated with early motherhood in all the five countries. Having secondary and above education reduced the odds of adolescent pregnancy by 67% (OR, 0.33; 95% CI, 0.21–0.54) in Kenya, by 65% (OR; 0.35,95% CI:0.19–0.62) in Tanzania, by 63% (OR, 0.37; 0.20–0.71) in Malawi, by 68% (OR; 0.32,95% CI: 0.16–0.87) in Uganda and 65% (OR, 0.35; 0.16–0.76) in Zambia compared to adolescents without a formal education.

**Table 4:** Multilevel analysis of the association between individual, household and community variables with adolescent pregnancy and early motherhood in five East African countries

Variables	Kenya aOR (95% CI)	Tanzania aOR (95% CI)	Malawi aOR(95%CI)	Uganda aOR(95%CI)	Zambia aOR(95%CI)
Age in years					
15 (ref)					
16	2.13(1.35–3.35)**	2.21(1.21–4.01)**	2.25(1.47–3.43)**	1.61(0.94–2.78)	1.63(1.05–2.53)**
17	5.27(3.38–8.21)**	6.14(3.36–11.19)**	5.89(3.86–8.98)**	3.50(2.04–6.02)**	3.53(2.28–5.47)**
18	9.88(6.38–15.30)**	10.35(5.72–18.72)**	9.31(6.14–14.11)**	9.20(5.33–15.88)**	6.55(4.27–10.05)**
19	20.43(12.96–32.18)**	26.09(13.80–49.31)**	18.02(11.6–28.1)**	14.57(8.28–25.63)**	16.56(10.52–26.07)**
Education					
No education (ref)					
Primary education	1.04(0.67–1.61)	0.76(0.46–1.24)	0.92(0.54–1.67)	0.57(0.22–1.47)	0.49(0.23–1.05)
Secondary and above	0.33(0.21–0.54)**	0.35(0.19–0.62)**	0.37(0.20–0.71)**	0.32 (0.16–0.87)**	0.35 (0.16–0.76)**
Age at first sex					
No sex	0.18 (0.11–0.29)	1.00 (−)	1.00 (−)	1.00 (−)	0.22(0.14–0.34)**
5–14	4.27 (2.41–7.56)**	12.28(6.48–23.30)*	5.03 (3.38–7.49)**	5.52(3.52–8.67)**	4.01(2.51–6.63)**
15–17	3.24(1.91–5.40)**	4.81(2.78–8.30)**	2.81(2.02–3.92)**	3.73(2.54–5.47)**	3.21(2.13–4.96)**
18–19 (ref)					
Exposure to Media (ref = none)					
One of the 3 sources	1.04(0.81–1.34)	0.95(0.69–1.30)	1.01(0.80–1.26)	0.86(0.67–1.10)	0.79(0.61–1.02)
Two of the 3 sources	0.78(0.56–1.08)	0.87(0.56–1.34)	0.66(0.45–0.95)*	0.59(0.40–0.88)**	0.58(0.43–0.80)**
All three sources	0.57(0.34–0.95)**	0.70(0.34–1.33)	0.52(0.25–1.00)	0.36(0.18–0.72)**	0.44(0.28–0.67)**
Residence (RC = urban)					
Rural	1.67(0.90–1.53)	1.04(0.67–1.58)	0.88(0.62–1.26)	1.12(0.77–1.62)	0.98(0.71–1.34)
Wealth (ref. = poorest)					
Poorer	0.88(0.64–1.20)	1.15(0.75–1.75)	0.67(0.49–1.05)	1.01(0.70–1.42)	1.10(0.75–1.56)
Middle	0.86(0.61–1.22)	0.85(0.55–1.29)	0.82(0.59–1.13)	0.83(0.56–1.24)	0.82(0.55–1.22)
Richer	0.59(0.38–0.91)*	0.75(0.47–1.18)	0.80(0.57–1.13)	0.54(0.35–0.84)**	0.63(0.39–1.00)
Richest	0.32(0.18–0.57)**	0.41(0.23–0.74)**	0.46(0.32–0.68)**	0.39(0.23–0.68)**	0.47(0.26–0.83)**
R/ship to head of HH					
Daughter (ref)					
Spouse/head	15.89(11.55–22.09)**	4.19(2.77–6.33)**	4.91(3.79–6.37)**	4.62(3.39–6.29)**	14.79(11.55–18.63)**
Living with relatives or others	2.00 (1.59–2.52)**	1.07(0.78–1.469)	1.23(0.99–1.54)*	1.79(1.37–2.33)**	1.01(0.81–1.26)
Sex of household head					
Male (ref)					
Female	0.87(0.71–1.07)	0.85(0.61–1.17)	1.08(0.88–1.34)	0.91(0.72–1.16)	1.06(0.84–1.34)
Comm. Level poverty (Ref = low)					
High	0.82 (0.61–1.10)	1.33(0.85–2.07)	1.09 (0.85–1.39)	0.70(0.49–0.94)*	0.83(0.59–1.17)
Comm. level education (ref = Low)					
High	1.01(0.78–1.30)	0.77(0.50–1.19)	1.12(0.84–1.47)	0.91(0.67–1.24)	1.25 (0.88–1.77)
ICC (empty model)	0.123	0.215	0.074	0.072	0.152
ICC (full model)	0.121	0.079	0.051	0.046	0.029
n	5820	2904	5263	4263	3675
Cluster	1489	494	802	626	712

* significant at *P* < 0.05

** significant at *P* < 0.01

Household economic status was another factor significantly associated with early motherhood. The results indicate that adolescents in the richest household quintile in Kenya, Tanzania, Malawi, Uganda and Zambia had 68% (OR; 0.32,95% CI:0.18–0.57), 59% (OR; 0.41,95% CI:0.23–0.74), 54% (OR, 0.46; 0.32–0.68), 61% (OR; 0.39,95% CI: 0.23–0.68) and 53% (OR, 0.47; 0.26–0.83) lower odds of early motherhood respectively compared to adolescents in the poorest quintile in the five countries. The association was stronger in Kenya and Uganda where adolescents in both the richer and richest wealth quintiles had significantly lower odds of adolescent pregnancy.

Other factors associated with adolescent pregnancy in this study were exposure to media and family structure. In Kenya and Zambia, adolescents with exposure to three or more sources of media were 43% (OR; 0.57, 95% CI: 0.34–0.95) and 56% (OR; 0.44, 95% CI: 0.28–0.67) less likely to experience early motherhood compared to those with no exposure to any media sources respectively. With regards to family structure or living arrangement, married adolescents had higher odds of early motherhood in all the five countries. Similarly, adolescents living with relatives and non-relatives had significantly higher odds of early motherhood in Kenya and Uganda. The sex of the household head did not show a significant association with adolescent pregnancy in all five countries (Table 4). As expected, adolescents who initiate sexual intercourse early had higher odds of adolescent pregnancy before age 19. The two community level covariates – community level poverty and community level girls’ education - did not show significant association with adolescent pregnancy except in Uganda where a marginal association with adolescent pregnancy was observed.

## Discussion

This study examines the trends and determinants of adolescent pregnancy and early motherhood in five East African countries using data from the DHS. The evidence indicates that adolescent childbearing is common in these countries ranging from 18% in Kenya to 29% in Zambia and Malawi. Recent trends however show a decline in all five countries since 1990 albeit with inconsistent trends. Tanzania and Malawi for instance have experienced an upward trend in adolescent pregnancy during the latest surveys and this has caused several policy level conversations and debates in the last few years. However, the five countries also experienced greater improvements in some of the key determinants of adolescent sexual and reproductive health including educational attainment, exposure to media, knowledge and use of contraception. But nonetheless, there has been little change in age at sexual debut and age at first marriage.

The study identified several key determinants of adolescent pregnancy in the five countries. Overall, secondary and higher education and higher household wealth were associated with lower levels of adolescent pregnancy in all the five countries. This is widely accepted and is consistent with findings of previous studies [17, 24, 27]. Studies from developing and developed countries alike showed that higher education is protective against early and unintended pregnancies [18, 23, 24, 27]. As adolescents get greater access to education, the opportunities for avoiding early childbearing may improve due to increased knowledge and agency to prevent unintended pregnancies, delayed on set of sexual relations and marriage.

Socio-economic status, measured using the DHS wealth Index, is another major factor associated with adolescent pregnancy in all the five countries. Consistency in this socio-economic pattern across all the countries indicates the lack of policies and programs that prioritize the most marginalized groups and address inequities in access to sexual and reproductive health services and information for adolescents. Several studies have demonstrated the effects of poverty on adolescent pregnancy [17, 18, 22]. Systematic reviews of the determinants of adolescent pregnancy in Sub-Saharan Africa and European countries showed that low socio-economic status is one of the predisposing factors consistently associated with adolescent pregnancy [17, 18]. Although we did not find association between living in poorer community neighborhoods and adolescent childbearing, aside from in Uganda, the literature indicates that adolescents from families of low socio-economic status and living in poor neighborhoods are at greater risk of early and unintended pregnancies largely due to poverty and lower expectations of future economic success [21, 22].

Other factors associated with adolescent pregnancy in this study were exposure to media, family structure and age at first sex. Our study showed that access to two or three media sources were protective in Kenya, Malawi and Uganda but not in the other two countries indicating that the effects of mass media on adolescent pregnancy is mixed. The few studies that examined the effects of mass media on adolescent sexual behavior indicated that media could have positive and negative influences on adolescent sexual behavior based on the contents [28, 29].

Family structure is another important factor influencing adolescent pregnancy. While early marriage is the major cause of adolescent pregnancy and motherhood with over 60% of births to adolescents taking place in marriage, adolescents not living with their parents are at increased risk of early motherhood. This could potentially be due to the lack of parental support and guidance which exposes girls to early sexual debut and early motherhood. Our findings are in line with previous evidences from studies from various countries including systematic reviews that have found that adolescents who do not live with both biological parents are at increased risk of adverse sexual outcomes [17, 25].

As expected, early sexual debut is one of the predisposing factors for early motherhood in all the five countries. Several other studies also found that early sexual debut is a significant predictor of adolescent pregnancy, contraceptive nonuse and HIV infection [30–32]. For instance, a study of the determinants of adolescent pregnancy in urban, disadvantaged settings across five countries found that sexual initiation at or before age 14 is correlated with lack of contraceptive use and higher rates of HIV and STIs in Johannesburg and Baltimore cities [30]. Early sexual initiation may lead to higher sexual risk-taking behavior, such as having multiple partners and not using contraceptives, and early pregnancy [33, 34].

There are some important limitations to this study. Given the cross-sectional nature of our data, it was not possible to assess causality between individual, household and community level factors, and adolescent pregnancy. For example, education could be protective against early pregnancy, but pregnancy can also result in changes in education. In addition, our analysis is limited to looking at births and current pregnancies and does not capture adolescent pregnancies that end in miscarriage, abortion and stillbirth. Context and characteristics could be different for adolescent pregnancies ending in miscarriage or abortion. Recent studies from Uganda and Ethiopia showed that close to one in six adolescent pregnancies end in induced abortion [35, 36].

This analysis shows that more needs to be done to reduce adolescent pregnancy and early motherhood in these countries. As the 2016 Lancet Commission on Adolescents emphasized, investments in adolescent health and wellbeing will not only transform the lives of girls and boys around the world, but will also generate high economic returns, especially in low income countries [13]. Existing evidences on what works in reducing adolescent pregnancy show that sexual and reproductive health education, counselling and contraception provision are effective in increasing adolescent’s knowledge of sexuality and health, contraceptive use and decreasing adolescent pregnancy [12, 37]. However, such policies are either poorly implemented or nonexistent in these countries. Only few of the countries have adolescent sexual and reproductive health strategies as well as sexuality education programs. Accelerated implementation of existing policies are needed to promote access to and uptake of adolescent sexual and reproductive health services including programs that promote community acceptance and demand-generation activities that reduce barriers to uptake of SRH services by adolescents [15, 16].

This study also highlights the importance of considering multi-sectoral approaches to address adolescent pregnancy. Initiatives to expand girl’s education and reduce household poverty are also important in improving adolescent sexual and reproductive health. Broader development programs that have positive impacts on girls educational and employment opportunities, may potentially influence their agency and decision-making around if and when to have children. Further study is required to understand the effects of media exposure including social media and contextual community level factors on adolescent pregnancy and early motherhood.

## Abbreviations

DHS: Demographic and Health Surveys
FP: Family planning
HIV: Human Immuno-deficiency Virus
MOH: Ministry of Health
SSA: Sub-Saharan Africa
STIs: Sexually Transmitted Infections
WHO: World Health Organization
